# “Choosing Wisely” Imaging Recommendations: Initial Implementation in New England Emergency Departments

**DOI:** 10.5811/westjem.2017.1.32677

**Published:** 2017-03-08

**Authors:** Ali S. Raja, Arjun K. Venkatesh, Nathan Mick, Cristopher P. Zabbo, Kohei Hasegawa, Janice A. Espinola, Jane C. Bittner, Carlos A. Camargo

**Affiliations:** * Massachusetts General Hospital, Department of Emergency Medicine, Boston, Massachusetts; † Harvard Medical School, Department of Emergency Medicine, Boston, Massachusetts; ‡ Yale University School of Medicine, Department of Emergency Medicine and Center for Outcomes Research and Evaluation, New Haven, Connecticut; § Tufts University School of Medicine, Maine Medical Center, Department of Emergency Medicine, Medford, Massachusetts; ¶ Kent Hospital, Department of Emergency Medicine, Warwick, Rhode Island

## Abstract

**Introduction:**

In June 2016, the American College of Emergency Physicians (ACEP) Emergency Quality Network began its *Reduce Avoidable Imaging Initiative*, designed to “reduce testing and imaging with low risk patients through the implementation of *Choosing Wisely r*ecommendations.” However, it is unknown whether New England emergency departments (ED) have already implemented evidence-based interventions to improve adherence to ACEP *Choosing Wisely* recommendations related to imaging after their initial release in 2013. Our objective was to determine this, as well as whether provider-specific audit and feedback for imaging had been implemented in these EDs.

**Methods:**

This survey study was exempt from institutional review board review. In 2015, we mailed surveys to 195 hospital-affiliated EDs in all six New England states to determine whether they had implemented *Choosing Wisely*-focused interventions in 2014. Initial mailings included cover letters denoting the endorsement of each state’s ACEP chapter, and we followed up twice with repeat mailings to non-responders. Data analysis included descriptive statistics and a comparison of state differences using Fisher’s exact test.

**Results:**

A total of 169/195 (87%) of New England EDs responded, with all individual state response rates >80%. Overall, 101 (60%) of responding EDs had implemented an intervention for at least one *Choosing Wisely* imaging scenario; 57% reported implementing a specific guideline/policy/clinical pathway and 28% reported implementing a computerized decision support system. The most common interventions were for chest computed tomography (CT) in patients at low risk of pulmonary embolism (47% of EDs) and head CT in patients with minor trauma (45% of EDs). In addition, 40% of EDs had implemented provider-specific audit and feedback, without significant interstate variation (range: 29–55%).

**Conclusion:**

One year after release of the ACEP *Choosing Wisely r*ecommendations, most New England EDs had a guideline/policy/clinical pathway related to at least one of the recommendations. However, only a minority of them were using provider-specific audit and feedback or computerized decision support. Few EDs have embraced the opportunity to implement the multiple evidence-based interventions likely to advance the national goals of improving patient-centered and resource-efficient care.

## INTRODUCTION

In 2013, the American College of Emergency Physicians (ACEP) published 10 evidence-based *Choosing Wisely®* recommendations for emergency department (ED) use of diagnostic tests and treatments,^1^ which patients and their providers were encouraged to discuss in order to reduce low-value care. Five of these focused on high-cost imaging. Since the publication of the *Choosing Wisely* recommendations, a number of tools, including clinical pathways,^2^ computerized decision support (CDS),^3^ and provider-specific audit and feedback^4^ have focused on improving emergency physicians’ adherence to evidence-based imaging guidelines.

In June 2016, ACEP’s Emergency Quality Network (E-QUAL) began its *Reduce Avoidable Imaging Initiative*^5^ as part of the Centers for Medicare and Medicaid Services (CMS) Transforming Clinical Practice Initiative, designed to “reduce testing and imaging with low risk patients through the implementation of *Choosing Wisely r*ecommendations.” The initiative is a laudable endeavor meant to emphasize the many tools available to assist with adherence to these recommendations. However, there are sparse data on whether EDs have implemented any interventions to improve adherence to guidelines since their initial publication in 2013. Our objective was to investigate whether New England EDs implemented evidence-based interventions to improve adherence to ACEP *Choosing Wisely* recommendations after their release, and also whether provider-specific audit and feedback for imaging had been implemented in these EDs.

## METHODS

### Study Settings

This survey study was exempt from institutional review board review. In 2015 we used the 2012 National Emergency Department Inventory^6^ to identify 195 hospital-affiliated EDs in the six New England states of Connecticut, Maine, Massachusetts, New Hampshire, Rhode Island and Vermont. We mailed surveys to their ED directors to assess several structural and process measures of each ED including capabilities, characteristics and policies in 2014 (the year after the release of ACEP’s *Choosing Wisely* recommendations). These initial mailings included cover letters denoting the endorsement of each state’s ACEP chapter, and were followed up twice with repeat mailings to non-responders.

### Survey Questions

The survey included a total of 30 questions, of which two focused on interventions that EDs had implemented targeting the five *Choosing Wisely* imaging scenarios: head computed tomography (CT) studies (for minor traumatic brain injury [MTBI] and in asymptomatic adults with syncope); chest CT for low-risk pulmonary embolism (PE); lumbar spine magnetic resonance imaging (MRI) for atraumatic low back pain; and abdominal CT for renal colic. For each of the scenarios, respondents were asked dichotomous yes/no subquestions regarding whether they had implemented either a guideline/policy/clinical pathway and/or computerized decision support. Pediatric EDs and EDs without CT/MRI capability to which individual questions might not apply were asked to indicate “NA” (not applicable). “Guideline/policy/clinical pathway” and “computerized decision support” were not further defined to allow respondents flexibility in deciding which of their interventions fell into each category. Respondents were also asked whether their clinicians received provider-specific audit and feedback regarding use of advanced imaging (e.g., their utilization compared to other clinicians in their ED).

**Population Health Research Capsule**

What do we already know about this issue?

ACEP has recommended a number of tests that can be avoided as part of the Choosing Wisely initiative.

What was the research question?

Do EDs have guidelines, policies, pathways, decision support, or feedback regarding ACEP Choosing Wisely Initiatives?

What was the major finding of the study?

57% have guidelines, policies, or pathways, 40% have decision support, and only 28% provide feedback regarding Choosing Wisely.

How does this improve population health?

Adherence to Choosing Wisely requires more than just education - the use of the evidence-based tools we studied should improve adherence to Choosing Wisely.

### Outcome Measures and Statistical Analyses

The outcomes were the presence or absence of at least one reported intervention for each of the five *Choosing Wisely* imaging scenarios, as well as the use of provider-specific audit and feedback. Data analysis included descriptive statistics and a comparison of state differences using Fisher’s exact test.

## RESULTS

**Figure 1:**
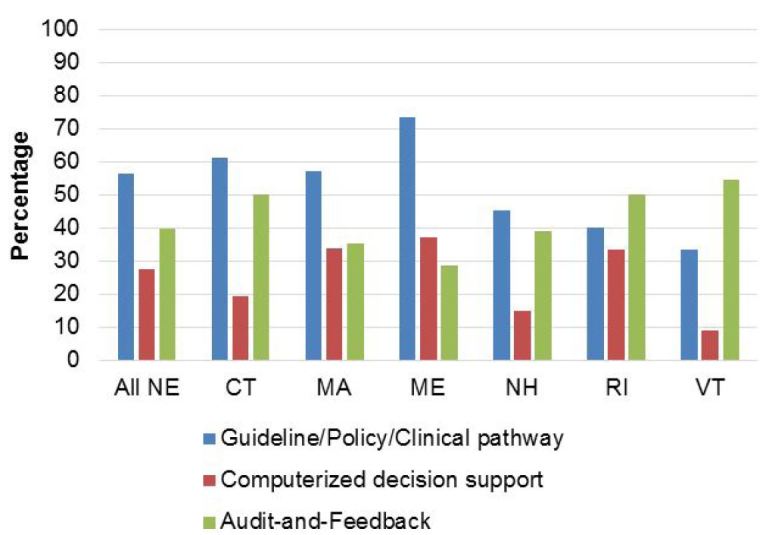
Interventions to reduce avoidable emergency department imaging in six New England states, in a study of the implementation of evidence-based *Choosing Wisely* recommendations. *NE,* New England; *CT,* Connecticut; *MA,* Massachusetts; *ME,* Maine; *NH,* New Hampshire; *RI,* Rhode Island; *VT,* Vermont.

Responses were received from 169/195 (87%) of New England EDs; all individual state response rates were >80%. Overall, 101 (60%) of responding EDs had implemented an intervention for at least one *Choosing Wisely* imaging scenario; a guideline/policy/clinical pathway (57% of EDs) was more frequently reported than CDS (28%) (Figure 1). In addition, 40% of EDs had implemented provider-specific audit and feedback, without significant interstate variation (range: 29–55%).

**Figure 2:**
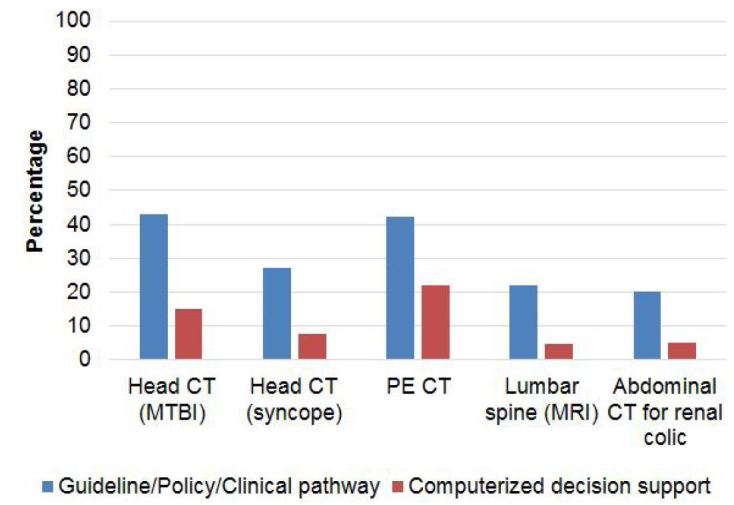
Interventions for *Choosing Wisely* clinical scenarios. *CT,* computed tomography; *MTBI,* mild traumatic brain injury; *PE,* pulmonary embolism; *MRI,* magnetic resonance imaging.

The most common interventions were for chest CT in patients at low risk of PE (47% of EDs), and head CT in patients with MBTI (45% of EDs) (Figure 2). By state, 63% of Maine EDs had implemented an intervention for head CT in patients with MTBI and 58% of Connecticut EDs had one for PE CT; interventions for the other three scenarios were observed less frequently (≤33% of responding EDs). Interventions were least commonly reported for abdominal CT for renal colic (21% of responding EDs); e.g., only one (8%) Vermont ED reported a policy for this scenario. There were no significant interstate differences in which *Choosing Wisely* targets had interventions implemented for them.

## DISCUSSION

One year after release of the ACEP *Choosing* Wisely recommendations, most New England EDs focused their interventions on only two imaging scenarios: patients with suspected PE and those with MTBI. While most EDs had a guideline/policy/clinical pathway related to at least one of the *Choosing Wisely* recommendations, only a minority had implemented CDS related to one of the recommendations. In addition, fewer than half of New England EDs were providing provider-specific audit and feedback about imaging utilization to their clinicians.

The *Choosing Wisely* recommendations are largely evidence-based and meant to target likely unnecessary and overused imaging studies. Translating these recommendations into clinical practice to reduce low-value care is the next needed step. A recent analysis of the effectiveness of the publication of several *Choosing Wisely* recommendations on outcomes (including head and lumbar spine imaging) found mixed results.^7^ The engagement of EDs in interventions beyond basic education through the 2016 *Reduce Avoidable Imaging Initiative* will be key to broad implementation of tools targeting the established *Choosing Wisely* targets.

For campaigns such as the E-QUAL *Reducing Avoidable Imaging Initiative* to succeed, understanding current ED interest and practice in imaging re-education is essential to guiding future efforts. From our data, it is evident that a number of EDs, at least in New England, are already focusing on reducing imaging in patients with suspected PE and suspected MTBI. Both conditions have a broader evidence base to guide imaging decisions, including widely disseminated ACEP clinical policies, likely making provider engagement in quality improvement (QI) easier. Conversely, clear evidence gaps remain for two of the other targets —CT for syncope and abdominal CT for renal colic —for which no clinical practice guidelines or many large clinical trials exist. In the case of lumbar spine MRI for back pain, which is supported by the *Choosing Wisely* campaigns of numerous medical specialty societies and clinical practice guidelines, lower rates of ED QI interventions may reflect that many of the guidelines are still based on expert consensus rather than evidence-based decision instruments. As national efforts such as E-QUAL continue to expand, resources must be dedicated to developing an evidence base and the associated clinical practice guidelines necessary to engender physician trust in recommendations to reduce imaging use historically considered necessary to exclude high-risk, life-threatening diagnoses.

Our work also demonstrates wide variability in the implementation of evidence-based QI strategies, with a notable lack of CDS and provider-specific audit and feedback. There is evidence that both of these interventions can improve the appropriateness of imaging use in the ED^3^,^4^,^8^,^9^ – specifically in the scenarios targeted by ACEP’s E-QUAL – and both tools should be considered by EDs looking to improve performance for *Choosing Wisely* recommendations. Poor adoption of CDS is surprising given the rapid adoption of electronic health records (EHR) in the ED as a result of the CMS Meaningful Use program. However, not all EHRs have easily-customized CDS capabilities, and ED staff may not have had the opportunity to readily implement CDS to address *Choosing Wisely* recommendations. As our survey preceded implementation of the 2014 Protecting Access to Medicare Act (PAMA) that mandates physician use of CDS, increased adoption of CDS is likely to be reflected in future surveys of EDs.

## LIMITATIONS

This study has two main limitations. The first is that it was conducted only in New England, and therefore results may not generalize nationally. We used a survey methodology that relies on self-reporting and we did not assess any potential differences in actual “on the ground” implementation. However, we have no reason to believe that respondents were untruthful, particularly as they were told in the survey instructions that no identifying information would be used and responses would be reported only in aggregate.

## CONCLUSION

Our assessment of initial ED efforts undertaken after publication of the *Choosing Wisely* recommendations shows broad interest in reducing avoidable imaging. However, the QI practices are largely limited to select interventions and certain clinical scenarios. Few EDs have embraced the opportunity to implement multiple evidence-based interventions likely to yield synergistic gains necessary for emergency care to advance the national goals of improving patient-centered and resource-efficient care.^10^
